# Acute Pulmonary Edema as a Cardiovascular Manifestation of Pheochromocytoma

**DOI:** 10.7759/cureus.33675

**Published:** 2023-01-12

**Authors:** Daniel Z Ng, Kyi Pyar Than Yu, Jeyanthy Rajkanna

**Affiliations:** 1 Department of Diabetes and Endocrinology, Peterborough City Hospital, Peterborough, GBR

**Keywords:** pulmonary edema, pheochromocytoma, acute pulmonary edema, stress induced cardiomyopathy, non-cardiogenic pulmonary edema, pheochromocytoma multisystem crisis, takotsubo cardioyopathy

## Abstract

Pheochromocytoma most commonly presents with the triad of paroxysms of headache, palpitations, and diaphoresis. Pheochromocytoma crisis, caused by a supra-physiological surge of catecholamine release, is an endocrine emergency that can present with various clinical manifestations. Acute pulmonary edema is one of the manifestations of pheochromocytoma crisis and can be either cardiogenic or non-cardiogenic. Here, we report cases of acute pulmonary edema of each type, related to pheochromocytoma crisis, which were presented to our district general hospital in 2020.

## Introduction

Pheochromocytoma is a rare neuroendocrine tumor, derived from chromaffin cells of the adrenal gland or relevant sympathetic nerves and ganglia. Estimates of the combined incidence of pheochromocytoma and the related paraganglioma are approximately 0.6 per 100,000 per year [1]. It is known as a rare cause of secondary hypertension. Diagnosis requires a high index of suspicion, given the broad spectrum of syndromes that produce symptoms of increased activation of the sympathetic nervous system. Aside from the long-term complications of poorly controlled hypertension, acute decompensated disease can occur in the form of pheochromocytoma crisis. This can range from hypertensive crises to rapidly progressive circulatory and multi-organ failure. Severe cardiorespiratory disease with acute pulmonary edema can also occur as a first presentation [2].

We present a report of two patients (cases 1 and 2) who had delayed diagnosis of pheochromocytoma after repeated admissions with a variety of symptoms and specific symptoms suggestive of acute pulmonary edema.

These cases were previously presented as poster at the Royal College of Physicians regional poster competition in East of England on September 15, 2021.

## Case presentation

Case 1

A 44-year-old woman presented to the emergency department three times over two months with severe dyspnea and cough productive of blood-stained sputum. She had previously experienced similar episodes, preceded by palpitations, over the last five months. Past medical history included intermittent palpitations and chest tightness since 2017, and anxiety and depression over four years.

**Figure 1:**
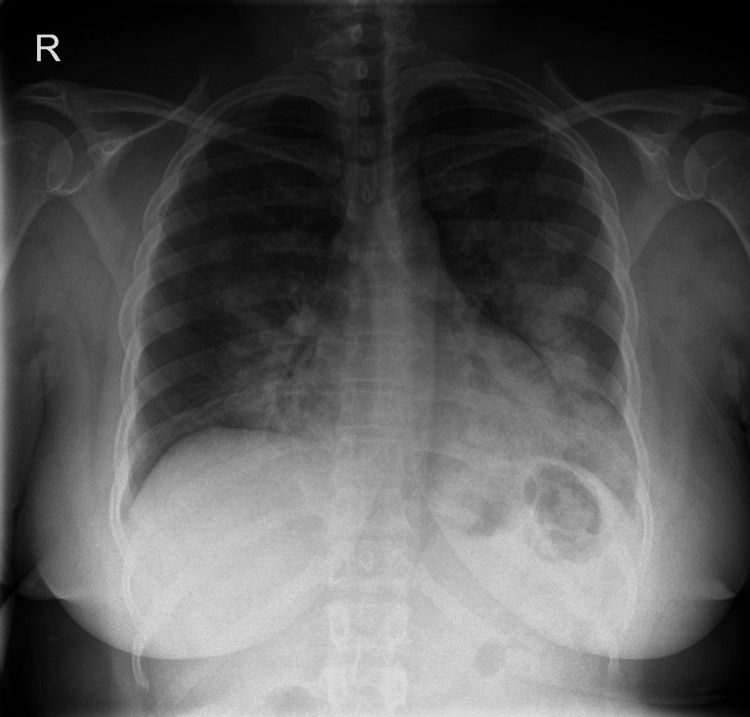
Chest radiograph demonstrating bilateral pulmonary infiltrates (case 1).

On her first admission, she was treated as pulmonary embolism while awaiting a pulmonary angiogram. Chest radiography showed confluent airspace opacification over both lung fields (Figure 1). Her SARS-CoV-2 polymerase chain reaction (PCR) test was negative and she was discharged on an oral anticoagulant while awaiting a computed tomographic pulmonary angiography (CTPA).

**Figure 2:**
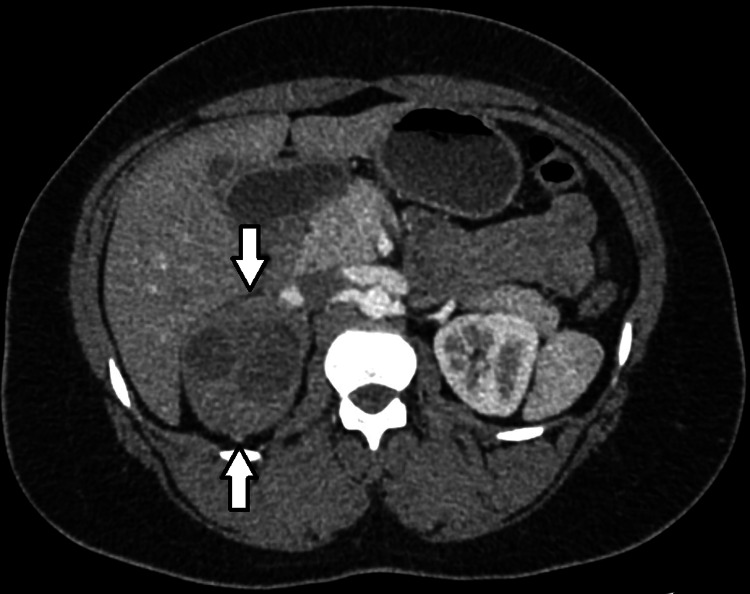
CT abdomen with contrast (case 1) - right-sided mixed semi-cystic mass (white arrows) lying inferior to liver and superior to but not arising from the right kidney. CT: computed tomography

Two weeks later, she attended the emergency department with the same symptoms. Chest radiography at this time was unremarkable and her recent CTPA showed no pulmonary embolism. However, it did identify an unexpected partially visualized right-sided abdominal mass. She had received a contrast-enhanced computed tomography (CT) of her abdomen which showed a large 6.5 × 7.3 × 6.9 cm right adrenal mass for which she was referred to the endocrine outpatient clinic (Figure 2). She was discharged with oral antibiotic.

**Figure 3:**
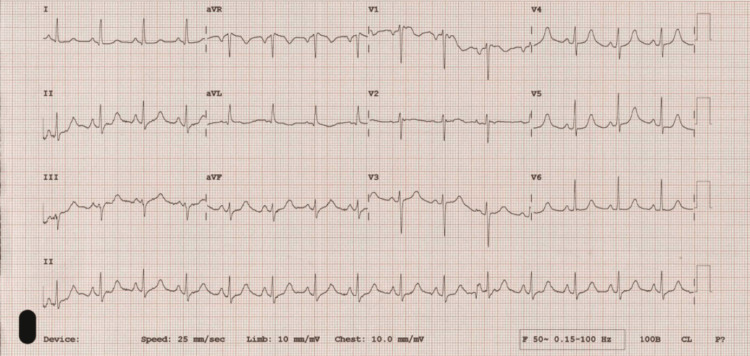
ECG (case 1) - normal sinus rhythm, no evidence of ischemia. ECG: electrocardiogram

She presented for the third time a further 10 days later. At presentation, she was tachypneic and sweaty, with a pulse rate of 150/min, a blood pressure of 91/50 mmHg, and peripheral oxygen saturation of 65% on air. She had ongoing hemoptysis. Widespread bilateral crackles were audible on lung auscultation. Heart sounds were normal, and no ventricular gallop was heard. Her arterial blood gas showed type 1 respiratory failure and her N-terminal pro-B-type natriuretic peptide (NT pro-BNP) level was elevated at 779 pg/mL. Chest radiography showed massive pulmonary edema but a normal-sized heart. Following stabilization, an electrocardiogram (ECG) revealed sinus rhythm with no evidence of myocardial infarction (Figure 3).

She was initially admitted to the intensive care unit for oxygen therapy and continuous positive airway pressure (CPAP) ventilatory support for suspected acute pulmonary edema. Intravenous fluid resuscitation was used for intermittent low blood pressure. Hypertension was managed with intravenous infusions of hydralazine and phentolamine, but these were rapidly discontinued due to hypotension. The patient recovered sufficiently to be discharged to the ward after one day. Following her discharge to the ward, plasma and 24-hour urinary metadrenaline and normetadrenaline levels were performed and both were elevated (Table 1). An echocardiogram showed only mild basal septal left ventricular hypertrophy with ejection fraction of 65-70%.

**Table 1:** Urinary and serum metadrenaline and normetadrenaline level (case 1). Levels of plasma metadrenaline and normetadrenaline of >900 and >2500 pmol/L, respectively, represent a high likelihood of catecholamine-secreting tumor as advised by our lab.

Biochemical tests	Case 1
Urinary metadrenaline (<1.4 μmol/24 hours)	85.6 μmol/24 hours
Urinary normetadrenaline (<3.5 μmol/24 hours)	39.5 μmol/24 hours
Plasma metadrenaline (<600 pmol/L)	12,372 pmol/L
Plasma normetadrenaline (<1000 pmol/L)	10,813 pmol/L

**Figure 4:**
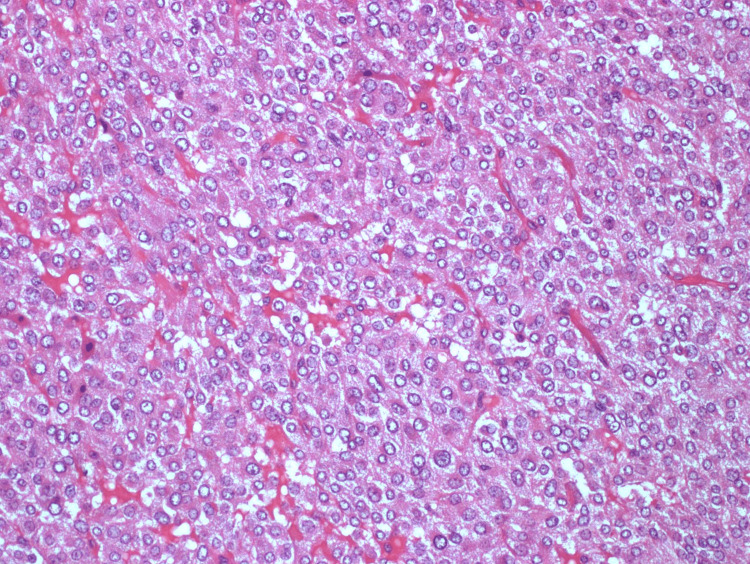
Hematoxylin and eosin stain of pheochromocytoma (case 1).

**Figure 5:**
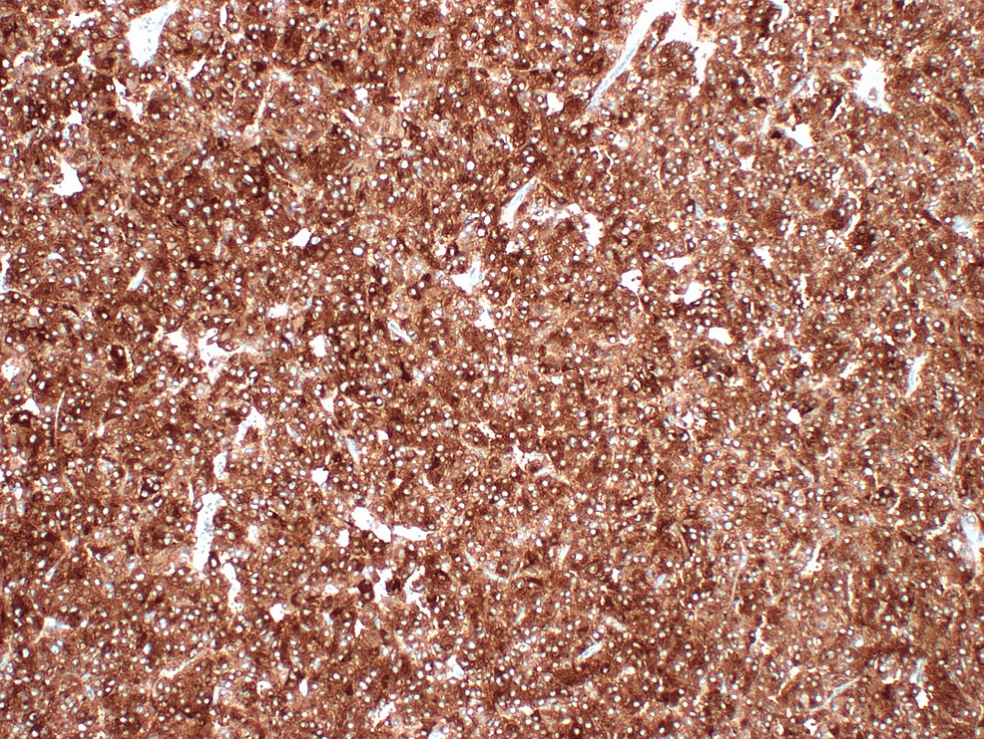
Synaptophysin immunohistochemistry staining of pheochromocytoma (case 1).

In view of her recurrent presentations with pulmonary edema, a past medical history of anxiety and recurrent palpitations, high urinary metadrenaline and normetadrenaline, and a large right adrenal mass, she was diagnosed with symptomatic pheochromocytoma. She was started on doxazosin for blood pressure control and the tumor was successfully resected without complications. Histological examination confirmed the diagnosis of pheochromocytoma (Figures 4, 5). Genetic testing for known pathological germline mutations was negative.

Case 2

A 51-year-old woman presented multiple times to the emergency department with headache, abdominal pain, nausea, and vomiting, followed by hypoxia and shortness of breath with a non-productive cough. Past medical history included hypertension, hypothyroidism, bicuspid aortic valve, and slightly dilated aortic root.

**Figure 6:**
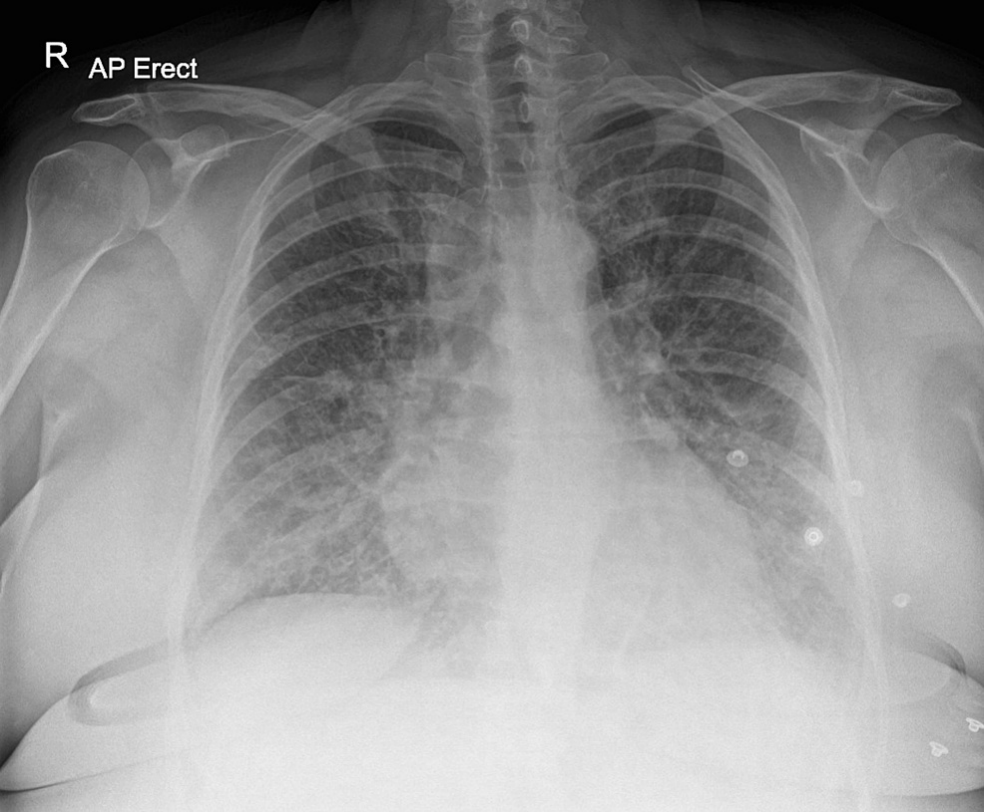
Chest radiograph demonstrating bilateral pulmonary infiltrates (case 2).

**Figure 7:**
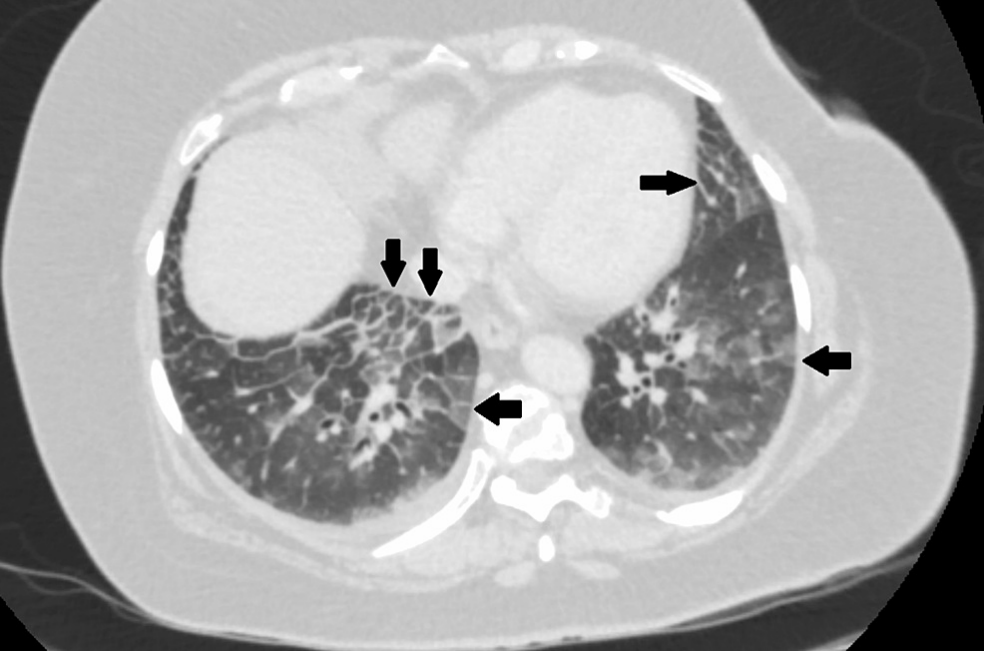
CT abdomen with contrast (case 2) - patchy crazy paving pattern opacification (arrows) seen on lung window at the lung bases. Crazy paving: ground-glass opacification with superimposed interlobular and intralobular septal thickening. CT: computed tomography

**Figure 8:**
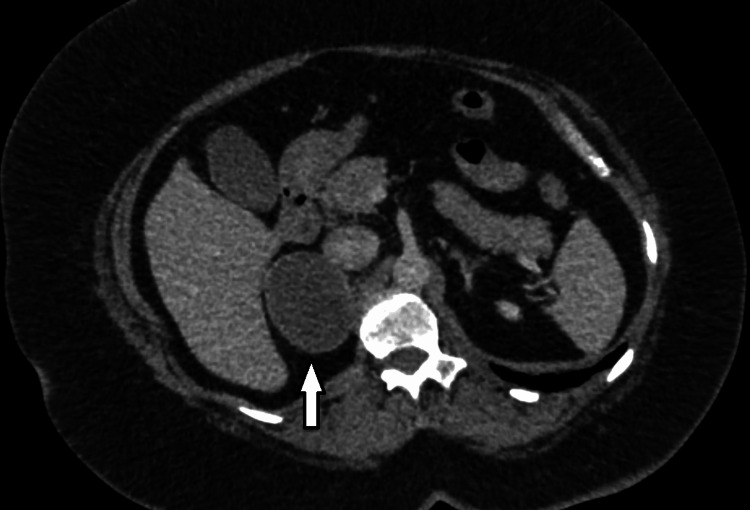
CT abdomen with contrast (case 2) - large right-sided cystic mass (arrow) seen below the liver but above the level of the kidney. CT: computed tomography

Her first two admissions occurred over two months and were evaluated with chest radiography (Figure 6) and CT of the abdomen with contrast (Figure 7). This showed lower lung crazy paving (Figure 7), and an incidental right-sided 58 mm adrenal mass (Figure 8). Her SARS-CoV-2 polymerase chain reaction (PCR) test was negative, and she was treated for suspected atypical pneumonia and discharged after successful weaning of ward-based oxygen therapy. Headache and vomiting were felt to be due to migraine.

One month after discharge from the hospital, she presented again with similar symptoms. She continued to vomit in the emergency department and was tachycardic at 111 beats per minute, and so received paracetamol, codeine, sumatriptan, ondansetron, and IV fluids. Her symptoms continued to worsen, and she was given metoclopramide and morphine. Unfortunately, she then developed a compensated lactic acidosis with a lactate of 6 mmol/L and type 1 respiratory failure, requiring CPAP.

**Figure 9:**
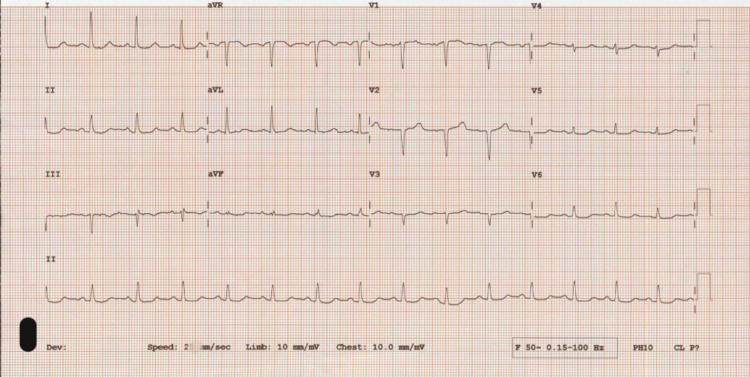
ECG (case 2) - sinus rhythm, Q-waves in V1-V3, subtle ST-depression in V4-V6, I, II, and aVF. ECG: electrocardiogram

Chest radiography demonstrated pulmonary edema, and furosemide was given, allowing for rapid wean off CPAP. ECG showed anterior Q waves with very subtle inferolateral ST depression, with a troponin-T rise from 6 ng/L to 184 ng/L. NT pro-BNP was slightly elevated at 502 pg/mL. CT aortogram ruled out dissection but did show some inflammatory change around the right upper kidney, raising the possibility of pyelonephritis. Urine and blood cultures were negative. Echocardiogram revealed mid-to-apical regional wall motion abnormalities (RWMAs) with reduced ejection fraction of 40-45%, which improved prior to discharge (Figure 9).

Following cardiology review, she was anticoagulated with fondaparinux and given dual antiplatelet therapy for suspected non-ST segment elevation myocardial infarction (NSTEMI), until inpatient computed tomographic coronary angiography (CTCA) revealed only distal posterior descending artery (PDA) stenosis. She received appropriate broad-spectrum antibiotics for suspected pyelonephritis and was discharged with a diagnosis of acute stress cardiomyopathy secondary to this.

Given the recurrent stereotyped presentations and right adrenal mass identified on abdominal imaging, plasma and 24-hour urinary metadrenaline and normetadrenaline levels were tested and found to be elevated (Table 2). She was subsequently referred to the endocrinology department, where she was reviewed urgently, and referred for surgical excision for a suspected right-sided pheochromocytoma. Her regular bisoprolol was tapered and exchanged for doxazosin. Thyroid function and the remainder of her adrenal screen including aldosterone-renin ratio, reproductive hormones, and urinary cortisol were unremarkable.

**Table 2:** Urinary and serum metadrenaline and normetadrenaline level (case 2). Levels of plasma metadrenaline and normetadrenaline of >900 and >2500 pmol/L, respectively, represent a high likelihood of catecholamine-secreting tumor as advised by our lab.

Biochemical tests	Case 2
Urinary metadrenaline (<1.4 μmol/24 hours)	1.5 μmol/24 hours
Urinary normetadrenaline (<3.5 μmol/24 hours)	4.1 μmol/24 hours
Plasma metadrenaline (<600 pmol/L)	1,732 pmol/L
Plasma normetadrenaline (<1000 pmol/L)	9,257 pmol/L

**Figure 10:**
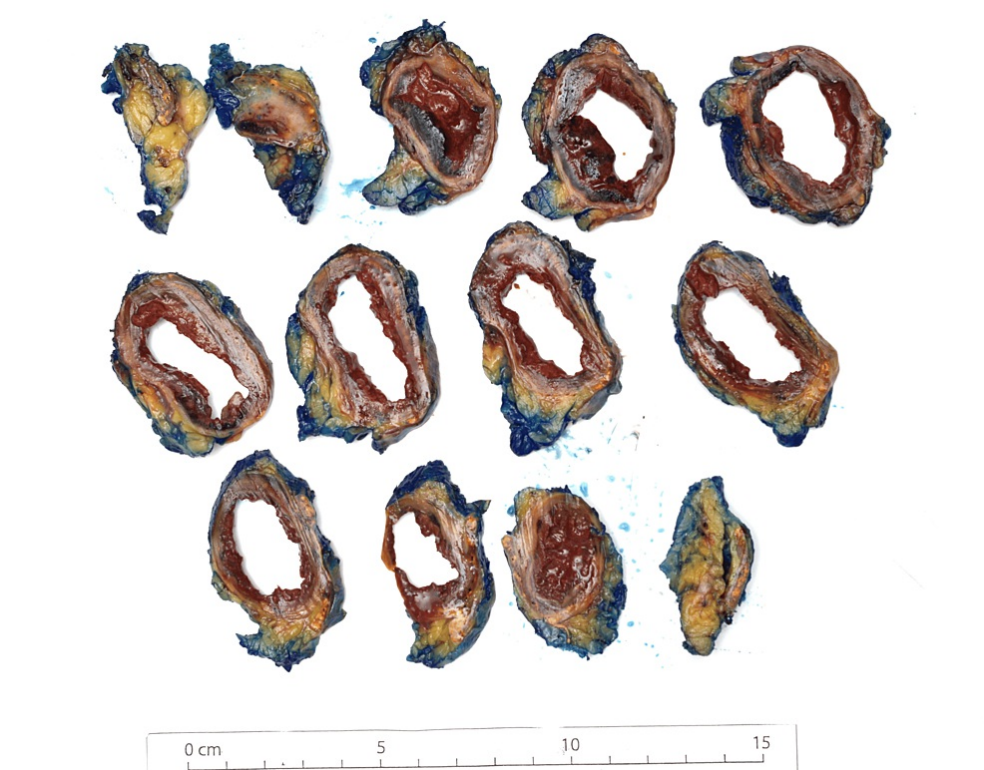
Gross image showing central cystic degeneration of pheochromocytoma (case 2).

**Figure 11:**
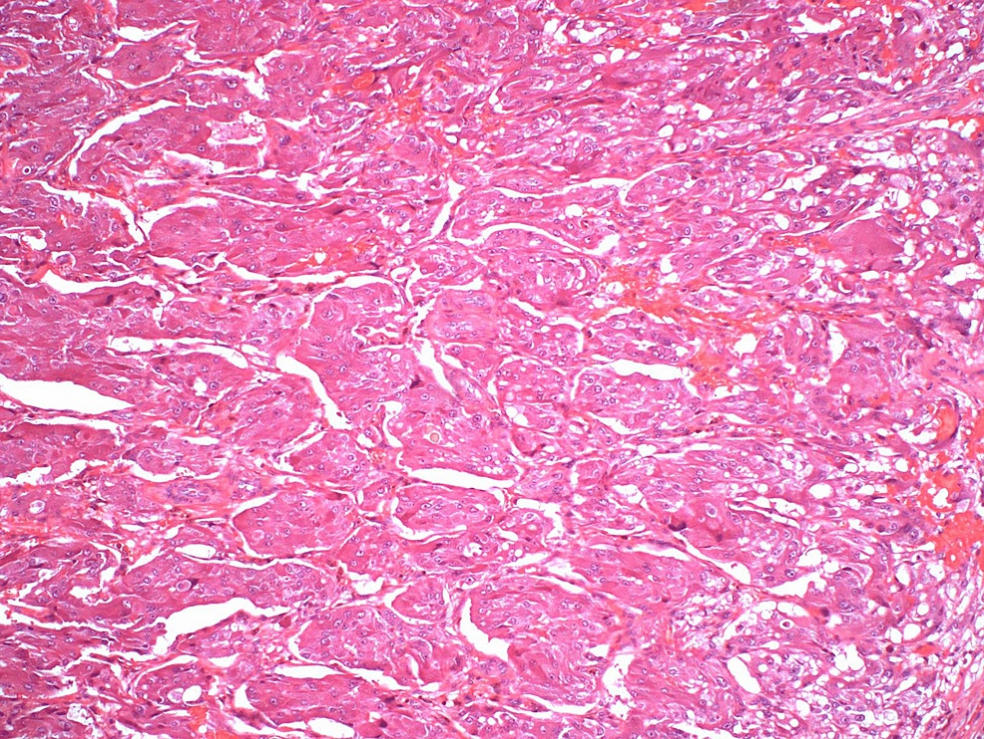
Hematoxylin and eosin staining of pheochromocytoma showing nested growth (case 2).

**Figure 12:**
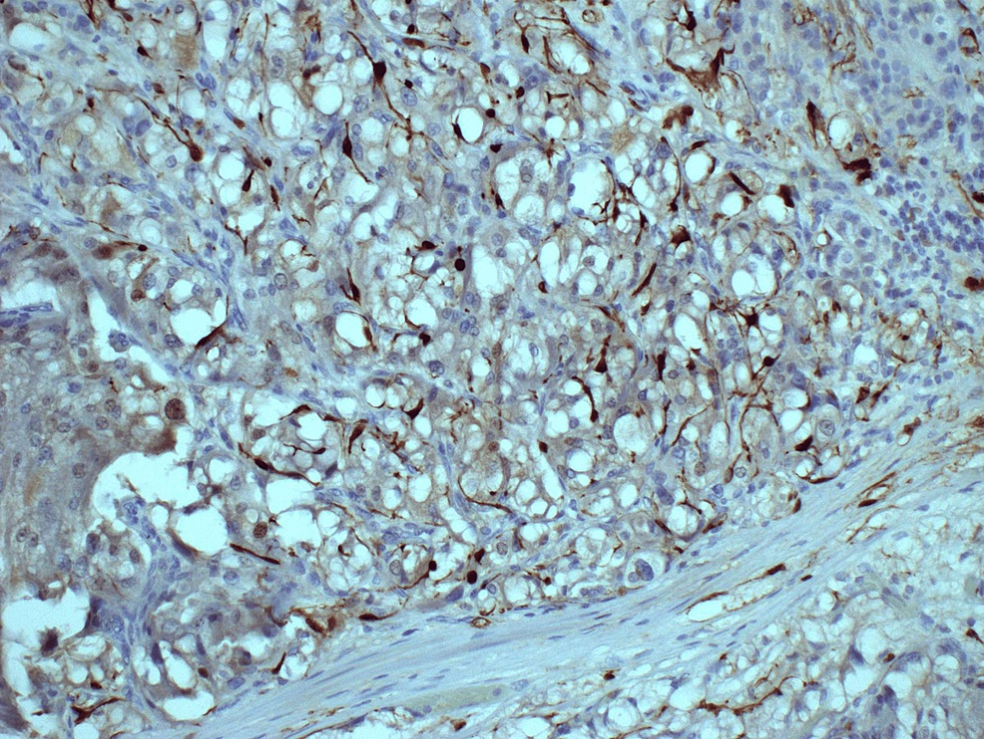
S-100 protein immunohistochemistry stain of pheochromocytoma showing partially retained sustentacular cell network (case 2).

**Figure 13:**
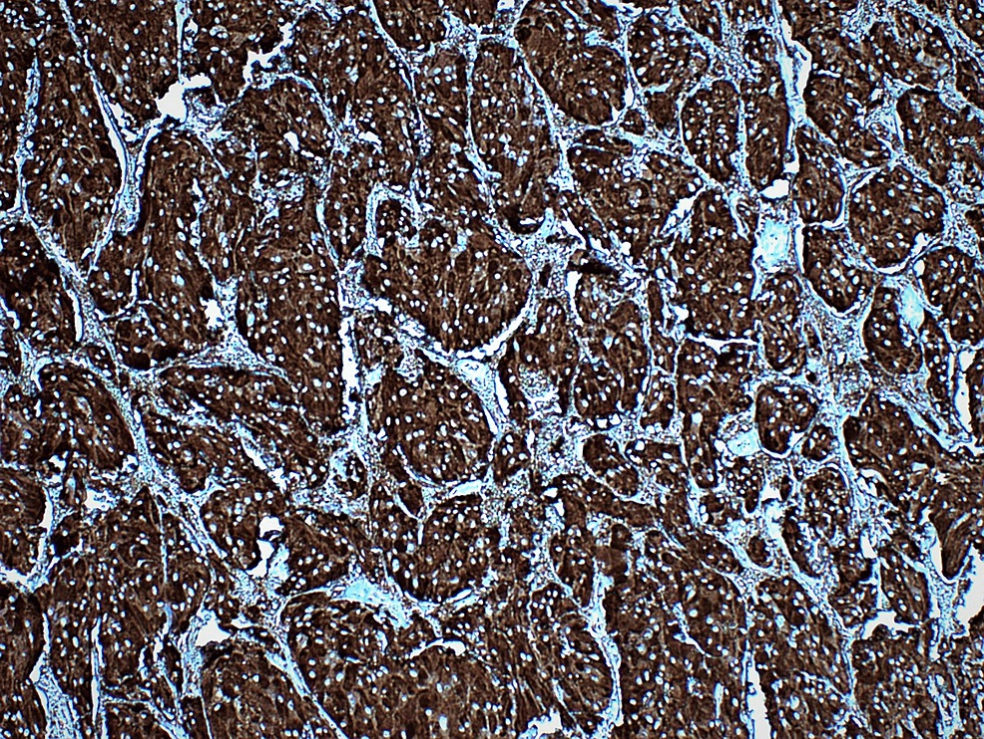
Chromogranin immunohistochemistry stain of pheochromocytoma (case 2).

Unfortunately, two weeks later she presented again to the emergency department with abdominal pain. Her blood pressure was labile, fluctuating between 230/141 and 76/30 mmHg. She was admitted to the intensive care unit for treatment of pheochromocytoma crisis, and then transferred to a tertiary center where she underwent an uneventful laparoscopic adrenalectomy. Histological examination confirmed a right adrenal gland pheochromocytoma with central infarction (Figures 10-13). Genetic profiling has revealed a variant of unknown significance in the succinate dehydrogenase complex flavoprotein subunit A (SDHA) gene.

## Discussion

The above two cases showed that pheochromocytoma can present with variable clinical manifestations. Acute pulmonary edema in pheochromocytoma can be both cardiogenic and non-cardiogenic. The most common cause is cardiogenic and may be caused by myocardial infarction, cardiomyopathy, or severe arrhythmia. Non-cardiogenic pulmonary edema is considered rare and De Leeuw et al. reported a case of pheochromocytoma presenting with non-cardiogenic pulmonary edema and proposed that occurred as a result of catecholamine-induced transient increase in pulmonary capillary pressure due to pulmonary vasoconstriction and altered pulmonary capillary permeability [3]. In addition, Sukoh et al. reported a case of non-cardiogenic pulmonary edema associated with pheochromocytoma and suggested a role for neutrophil-mediated lung injury, driven by the catecholamine surge [4].

Our patient in the first case presented on multiple occasions with dyspnea and hemoptysis which were first misdiagnosed as pulmonary embolism and COVID-19 infection. She was diagnosed with acute pulmonary edema on her final admission. The combination of anxiety disorder, recurrent episodes of pulmonary edema, and the findings on CTPA raised the suspicion of pheochromocytoma, and increased urinary metadrenaline and normetadrenaline levels confirmed the diagnosis. In her case, there were no features to suggest that pulmonary edema is secondary to a cardiac cause as her ECG revealed only sinus tachycardia and echocardiogram showed only mild basal septal left ventricular hypertrophy. Therefore, we concluded that acute pulmonary edema in her case could be non-cardiogenic, secondary to a surge in plasma catecholamines, leading to a transient increase in pulmonary capillary pressure and altered pulmonary capillary permeability.

Our patient in the second case presented on multiple occasions with episodes of hypoxia, and, in contrast to the first case, echocardiogram demonstrated reduced left ventricular systolic function. This was supportive of a diagnosis of cardiogenic pulmonary edema. Initial investigations were directed towards positively identifying a driver, such as rupture of underlying atherosclerotic coronary artery plaque or dissection involving coronary arteries, which were relatively unrevealing. Interestingly, left ventricular function showed improvement over the course of a week, and ejection fraction normalized by the point of discharge. As her initial CT aortogram had shown some inflammation around the kidney, she was treated for pyelonephritis, and it was proposed that she had developed stress cardiomyopathy secondary to pyelonephritis, rather than myocardial infarction. This would explain such a rapid improvement in left ventricular function, as well as acute pulmonary edema in the context of disproportionately mild coronary artery disease.

Stress cardiomyopathy, also known as Takotsubo cardiomyopathy or apical ballooning syndrome, is a recognized but uncommon complication of pheochromocytoma. It can present similarly to an acute coronary syndrome, but without significant obstructive coronary artery disease [5]. Catecholamine-driven cardiac impairment may arise from direct cardiotoxicity via myocardial catecholamine-receptor signalling, microvascular ischemia, and transient coronary artery vasospasm [5]. It has been suggested that all patients with suspected Takotsubo cardiomyopathy should be evaluated for underlying endocrine drivers, such as pheochromocytoma and thyrotoxicosis [6]. Our patient in the second case likely developed acute Takotsubo cardiomyopathy related to pheochromocytoma, with the inflammatory change around the upper pole of the right kidney more likely to represent non-specific changes related to her acute crisis.

## Conclusions

In conclusion, pheochromocytoma is a rare clinical condition and a diagnostic challenge. It should be considered in every patient presenting with intermittent palpitations, headache, and diaphoresis to achieve a timely diagnosis. Pheochromocytoma can present with various cardiovascular manifestations, including circulatory failure. It should be included in the differential diagnosis of acute pulmonary edema where no other obvious cause can be elicited. In addition, the diagnosis of Takotsubo cardiomyopathy should lead to an active search for pheochromocytoma.
